# Supplementary material for: HIV-1 Proviral Genome Engineering with CRISPR-Cas9 for Mechanistic Studies
Source: Viruses. 2024 Feb 13;16(2):287. doi: 10.3390/v16020287 (PMC10892460; doi:10.3390/v16020287)
Supplement: Supplementary file 1 [file viruses-16-00287-s001.zip › viruses-2792154-supplementary.pdf]

Supplementary Information

HIV-1 proviral genome engineering with CRISPR-Cas9 for mechanistic studies

Usman Hyder[†], Ashutosh Shukla[†,‡], Ashwini Challa and Iván D'Orso[*]

Department of Microbiology, The University of Texas Southwestern Medical Center, Dallas, TX 75390, USA;

[†] These authors contributed equally

[‡] Current address: Division of Digestive and Liver Diseases, The University of Texas Southwestern Medical Center, Dallas, TX 75390, USA;

*For correspondence: ivan.dorso@utsouthwestern.edu

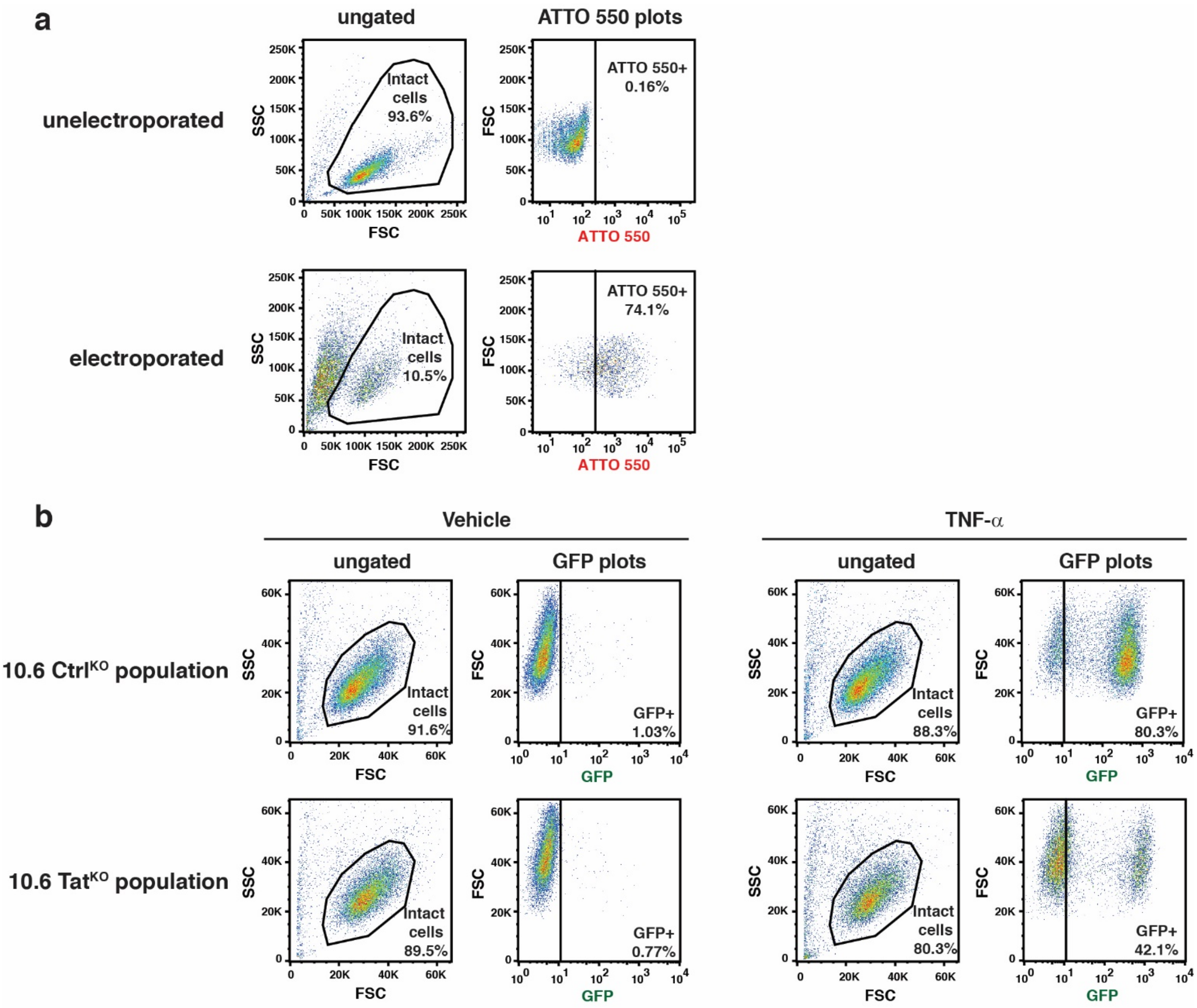

Supplementary Figure S1. J-Lat 10.6 Tat^{KO} cells can be made using a CRISPR-Cas9–based approach. (**a**) Raw flow cytometry plots (ungated cells and ATTO 550+ cells) for data in Figure 3a. (**b**) Raw flow cytometry plots (ungated cells and GFP+ cells) for data in Figure 3b.

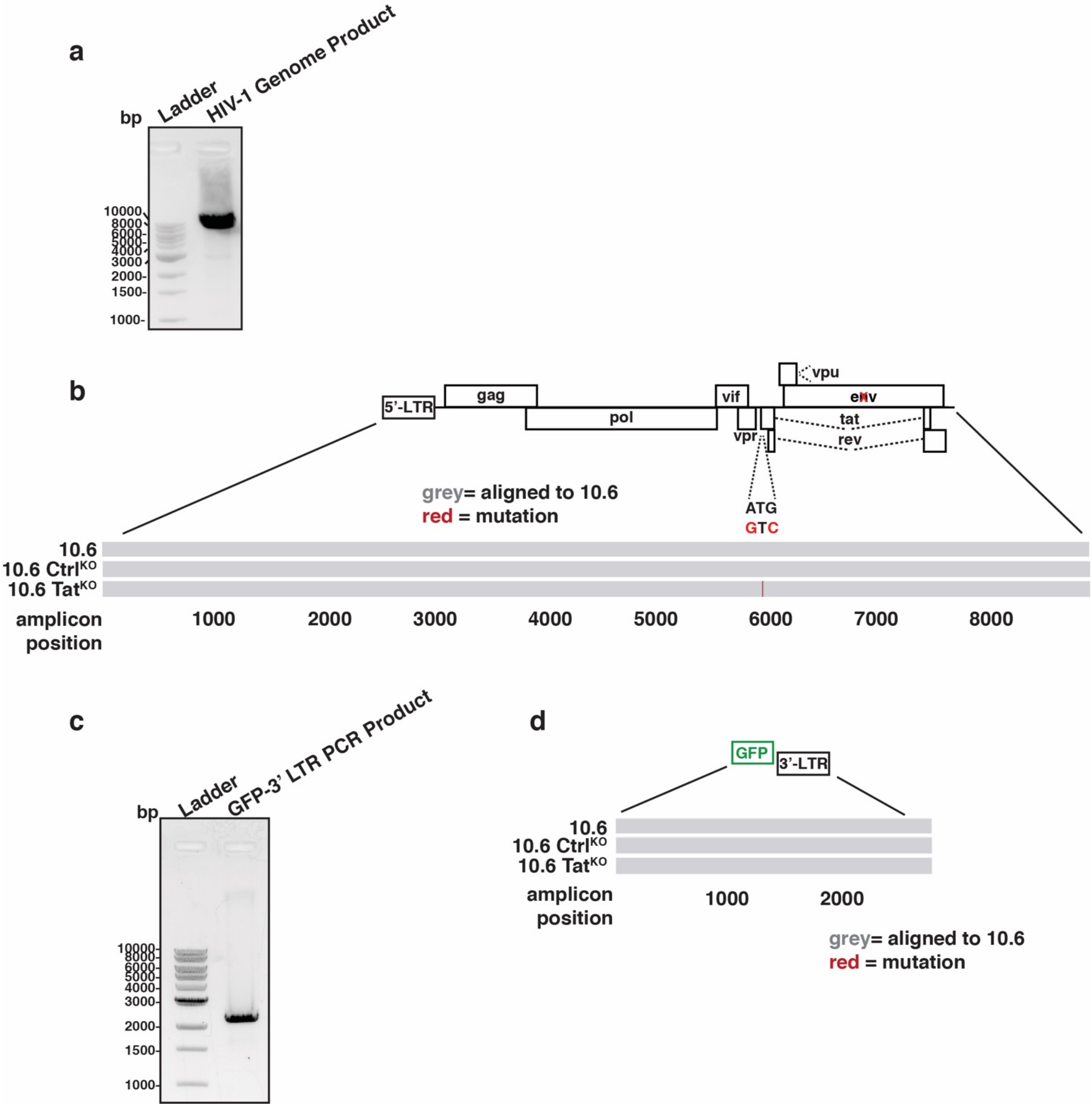

Supplementary Figure S2. HIV-1 genome sequencing in 10.6 Ctrl^{KO} and 10.6 Tat^{KO} clones. (**a**) Agarose gel showing the HIV-1 genome PCR product. Parental 10.6 cells are shown as an example. (**b**) Amplicon sequencing of the HIV-1 genome PCR products showing alignment between DNA obtained from 10.6, 10.6 Ctrl^{KO}, and 10.6 Tat^{KO} cells. (**c**) Agarose gel showing the GFP–3′ LTR PCR product. 10.6 parental cells are shown as an example. (**d**) Amplicon sequencing of the GFP–3′ LTR PCR products showing alignment between DNA obtained from 10.6, 10.6 Ctrl^{KO}, and 10.6 Tat^{KO} clones.

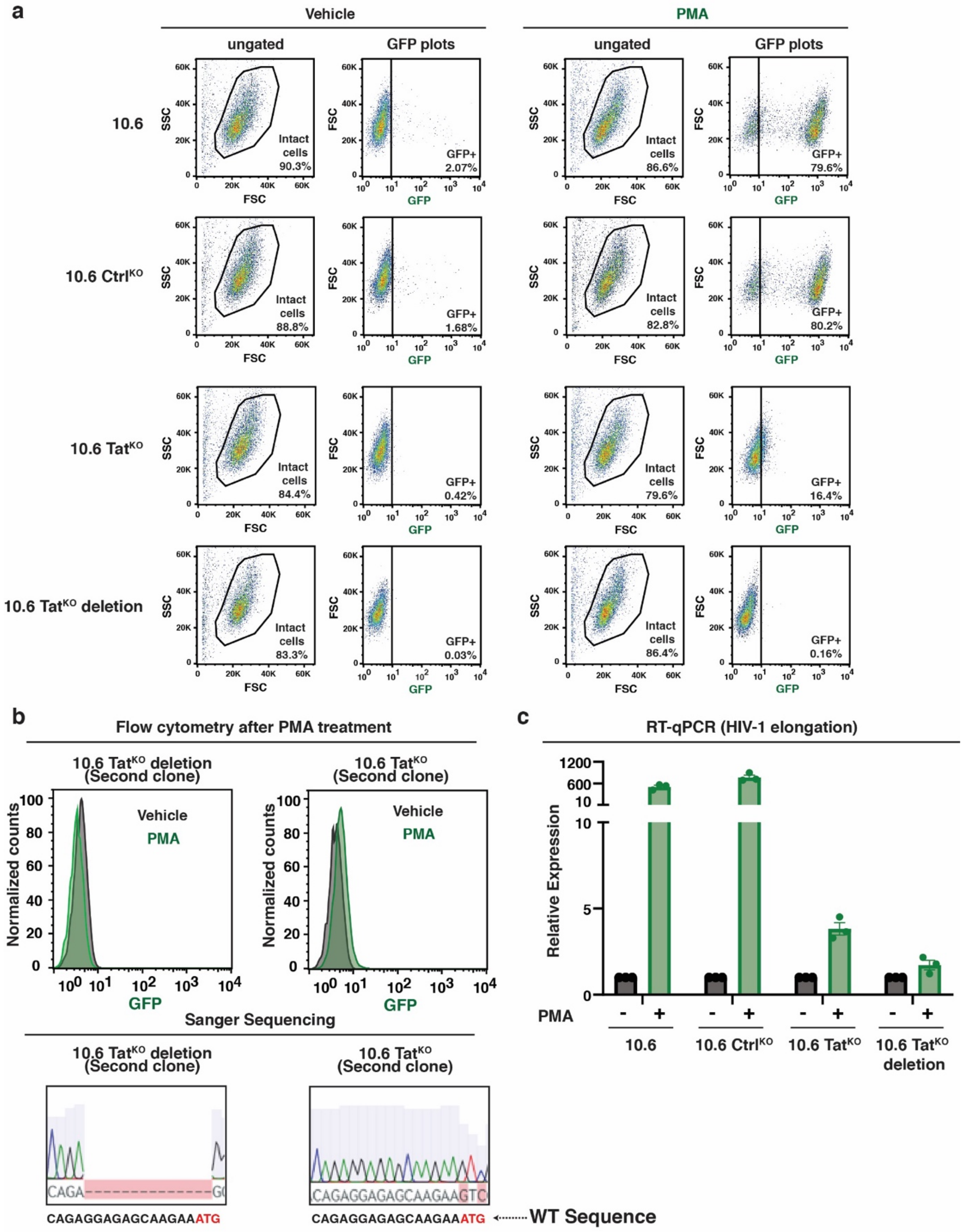

Supplementary Figure S3. Precise provirus genomic mutations and large deletions lead to differences in HIV-1 transcription induction upon cell stimulation. (**a**) Raw flow cytometry plots (ungated cells and GFP+ cells) for data in Figures 4a-b. (**b**) Flow cytometry histograms of the indicated clones treated with vehicle DMSO or PMA. The Sanger sequencing data for each clone is indicated below. (**c**) RT-qPCR data for HIV-1 elongation in 4 cell lines (10.6, 10.6 Ctrl^{KO}, 10.6 Tat^{KO}, and 10.6 Tat^{KO} deletion) –/+ 16 h PMA. n=3, –/+ SEM.

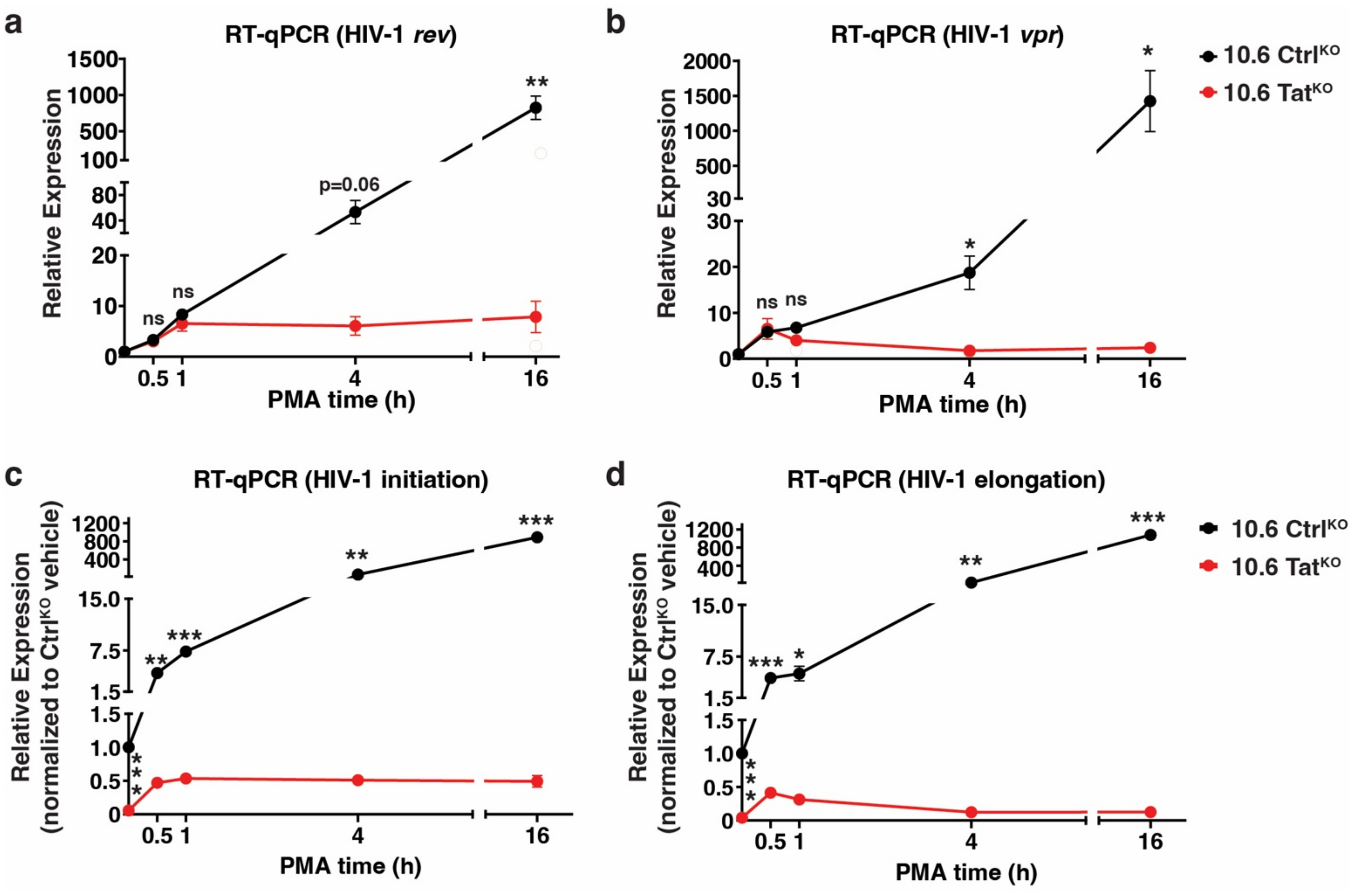

Supplementary Figure S4. Tat sustains enhanced HIV-1 expression upon cell stimulation. (**a-b**) RT-qPCR for HIV-1 *rev* (**a**) and *vpr* (**b**) in 10.6 Ctrl^{KO} and 10.6 Tat^{KO} cell lines across the PMA time course. All data presented in these plots are normalized to the respective vehicle control for that cell line. Statistics are compared between 10.6 Ctrl^{KO} and 10.6 Tat^{KO} for each time point using an unpaired Student's t-test, n=3, –/+ SEM, ns = non-significant, * $p < 0.05$, ** $p < 0.01$. (**c-d**) RT-qPCR for HIV-1 initiation (**c**) and HIV-1 elongation (**d**) in 10.6 Ctrl^{KO} and 10.6 Tat^{KO} cell lines across the PMA time course. All data presented in these plots are normalized to the 0 time point of the 10.6 Ctrl^{KO} cell line. Statistics are compared between 10.6 Ctrl^{KO} and 10.6 Tat^{KO} for each time point using a unpaired Student's t-test, n=3, –/+ SEM, * $p < 0.05$, ** $p < 0.01$, *** $p < 0.001$.

Supplementary Table S1. Oligos used in this paper.

Number	Name	Sequence (5′-3′)	Usage	Modifications
	Tat ATG (10.6) crRNA oligo	CGACAGAGGAGAGCA AGAAA	Annealed to tracrRNA, ATTO™ 550 to create the gRNA	N-terminal AltR1 C-terminal AltR2
	HIV-1 10.6 Positive Donor oligo	G*A*CATAGCAGAATAG GCGTTACTCGACAGAG GAGAGCAAGAAGTCG AGCCAGTAGATCCTAG ACTAGAGCCCTGGAAG CATCC*A*G	HDR donor oligo	N-terminal Alt-R-HDR1 C-terminal Alt-R-HDR2 4 Phosphorothioate bonds (*)
	HIV-1 10.6 Negative Donor oligo	C*T*GGATGCTTCCAGG GCTCTAGTCTAGGATCT ACTGGCTCGACTTCTTG CTCTCCTCTGTCGAGTA ACGCCTATTCTGCTATG *T*C	HDR donor oligo	N-terminal Alt-R-HDR1 C-terminal Alt-R-HDR2 4 Phosphorothioate bonds (*)
3078	HIV-1_Tat_For	AGCCACACAATGAATG GACA	PCR	
3079	HIV-1_Tat_Rev	CAAACTTGGCAATGAA AGCA	PCR; Sanger sequencing	
3931	10.6_up 5′ LTR_F	CGTACTGGCTGGAGTA ATAGCT	PCR of HIV-1 genome	
3932	eGFP-N	CGTCGCCGTCCAGCTC GACCAG	PCR of HIV-1 genome	
3933	Frag-26-R-RC	CTGGCTGTGGAAAGAT ACCT	PCR of GFP and 3′ LTR	
3934	10.6_down 3′ LTR_R	GAATGCCCATTGCTTTG GGAA	PCR of GFP and 3′ LTR	
3076	HIV-1_elongation_FWD	GACGGTACAGGCCAGA CAAT	RT-qPCR ChIP-qPCR	
3077	HIV-1_elongation_REV	GATGCCCCAGACTGTG AGTT	RT-qPCR ChIP-qPCR	
1111	HIV-1_initiation_FWD	GCTTAAGCCTCAATAA AGCTTGCCTTGAG	RT-qPCR	
1112	HIV-1_initiation_REV	GTCCTGCGTCGAGAGA GCTCCTCTG	RT-qPCR	
3991	HIV-1_*rev*_FWD	TTCAGCTACCACCGCTT GAG	RT-qPCR	
3992	HIV-1_*rev*_REV	TATTTGAGGGCTTCCCA CCC	RT-qPCR	
3372	HIV-1_*vpr*_FWD	AGCCCCAGAAGACCA AGG	RT-qPCR	
3373	HIV-1_*vpr*_REV	TTGCCCTAAGCCATGG AG	RT-qPCR	
1868	*GAPDH*_FWD	GCAAATTCCATGGCAC CGT	RT-qPCR	

1869	*GAPDH*_REV	TCGCCCCACTTGATTTTGG	RT-qPCR	
9	*U6*_FWD	CTCGCTTCGGCAGCACATATAC	RT-qPCR	
10	*U6*_REV	GGAACGCTTCACGAATTTGCGTG	RT-qPCR	

Supplementary Table S2. Antibodies used in this paper.

Target	Company	Catalog Number	Assay (Dilution/time)
Tat	Abcam	ab43014	Western blot (1:500/ON)
Gag/p24	NIH HIV reagents Program	4121 (Mab to HIV-1 p24 (AG3.0))	Western blot (1:500/ON)
Actin Rhodamine	Bio-Rad	12004163	Western blot (1:10000/1 h)
Goat anti-mouse IgG-HRP	Santa Cruz Biotechnologies	sc-2005	Western blot (1:10000/1 h)
Donkey anti-rabbit IgG-HRP	Santa Cruz Biotechnologies	sc-2313	Western blot (1:10000/1 h)
RPB3 (Pol II)	Millipore	ABE999	ChIP-qPCR (5 µg/ON)
